# Navigating a Diagnostic Dilemma: A Case Report of Overlapping Presentation of Granulomatosis With Polyangiitis and Tuberculosis

**DOI:** 10.7759/cureus.84378

**Published:** 2025-05-19

**Authors:** Dinusha Gayathri, Udaya Ralapanawa, Inoka Shyamali, AMBD Alahakoon, GMC Weerasooriya

**Affiliations:** 1 General Medicine, Postgraduate Institute of Medicine, University of Colombo, Colombo, LKA; 2 General Medicine, Teaching Hospital Peradeniya, Peradeniya, LKA; 3 Internal Medicine, University of Peradeniya, Peradeniya, LKA; 4 Medicine, Rockhampton Base Hospital, Rockhampton, AUS; 5 Medicine, National Hospital of Sri Lanka, Colombo, LKA; 6 Hematology, Postgraduate Institute of Medicine, University of Colombo, Colombo, LKA

**Keywords:** asian countries, diagnosis, dilemma, granulomatosis with polyangiitis, tuberculosis

## Abstract

Granulomatosis with polyangiitis (GPA) and tuberculosis (TB) present a diagnostic conundrum due to their similar clinical manifestations, histopathological features, and the presence of positive antineutrophil cytoplasmic antibodies (ANCAs) in both conditions. We present the case of a 54-year-old patient who was initially evaluated for TB but developed a vasculitic rash during the course of the illness, suggesting an alternative diagnosis. Further evaluation revealed positive cytoplasmic ANCA (C-ANCA) and pulmonary nodules with cavitations on chest imaging, shifting the diagnosis toward GPA. The diagnosis of GPA was confirmed based on the EULAR/ACR (European League Against Rheumatism/American College of Rheumatology) 2022 classification criteria. Disease remission was induced with steroid pulse therapy and intravenous cyclophosphamide. Azathioprine was used for maintenance therapy. The patient made a remarkable recovery with treatment. We discuss this case due to the scarcity of reported cases in Sri Lanka to provide insight into the diagnostic approach for patients presenting with similar clinical phenotypes. Although TB is more common than GPA in Asian countries, few cases have been reported with overlapping features of both diseases. In light of this diagnostic dilemma, clinicians are faced with significant challenges in accurately distinguishing between the two diseases, which could lead to delays in the establishment of appropriate treatment and management. Therefore, we highlight the importance of considering GPA as a differential diagnosis for TB, even in Asian countries.

## Introduction

The diagnosis of GPA, formerly known as Wegener's granulomatosis, can pose a diagnostic challenge with tuberculosis (TB) because of overlapping features between the two diseases. GPA is a rare disease and is more prevalent among Caucasians [1,2]. Its prevalence in the United States is around 3 in 100,000 persons [3]. The annual incidence of GPA among Asians ranges from 0.37 to 2.1 cases per million population [4]. Incidence of GPA is equal between males and females, with a peak incidence in the fourth or fifth decade of life.

There are overlapping clinical manifestations of GPA and TB at the onset, including a prodrome of constitutional symptoms, such as fever and malaise, followed by common respiratory symptoms such as cough, hemoptysis, and systemic manifestations such as arthralgia. Furthermore, histopathological changes reveal similar findings including granuloma formation.

Radiological similarities between TB and GPA, such as cavitary, nodular, and necrotic lung lesions, can complicate the diagnostic process. This overlapping symptomatology often makes it difficult to differentiate between the two conditions at the beginning. The autoantibodies commonly used to diagnose GPA can also be positive in TB, making it more difficult to confirm the diagnosis.

In case of an incorrect diagnosis of GPA, the immunosuppression may worsen the underlying TB. Conversely, a delayed diagnosis of GPA poses significant risks for long-term survival.

Despite the diagnostic challenges posed by the overlapping presentation of TB and GPA, it is crucial to make a correct diagnosis to start proper management and improve survival. Although GPA is rarer in Asian countries compared to Western countries, it should always be considered as a possibility in clinical presentations similar to TB.

## Case presentation

A 54-year-old male presented with progressive dyspnea over two months, which had worsened during the second month. He also developed a productive cough accompanied by three episodes of hemoptysis in the two weeks leading up to admission. Additionally, he has had significant loss of appetite and noted to have an 8 kg weight loss in the past month. The patient had no prior history of TB or known contact with TB-infected individuals. He is a smoker with three pack years. The patient denied chest pain, bleeding manifestations, or symptoms of heart failure.

Further inquiry revealed that the patient had been having intermittent low-grade fever for the past month, which was temporarily relieved by paracetamol. However, despite analgesics, he continued to experience persistent generalized malaise and arthralgia. There were no complaints of recurrent nasal discharge, crusting, congestion, perforation of the nasal septum, or hearing loss.

At the time of admission, the patient appeared emaciated with a body mass index of 18 kg/m^2^ and was pale. He was afebrile on admission. He did not have cervical, axillary, or inguinal lymphadenopathy, nor was there any evidence of vasculitic rashes. We did not notice any saddle nose deformity or inflamed nasal or ear cartilages. His respiratory rate was 18 breaths/minute. An auscultation of the lungs revealed bilateral coarse crepitations, and the trachea was located in the midline. The abdominal examination revealed no signs of organomegaly, and the remainder of the systemic examination was unremarkable.

The patient was investigated for suspected TB. His admission investigations are shown in Table 1.

**Table 1:** Basic investigations on admission

Investigation	Value	Reference range
Erythrocyte sedimentation rate	65 mm/hr	Less than 10 mm/hr
C-reactive protein	95 mg/L	0–5 mg/L
Full blood count		
White blood cell	18,500 u/L	4–10,000 u/L
Hemoglobin	9.2 g/dL	11–16 g/dL
Platelet count	537,000 u/L	150,000–450,000 u/L

**Figure 1:**
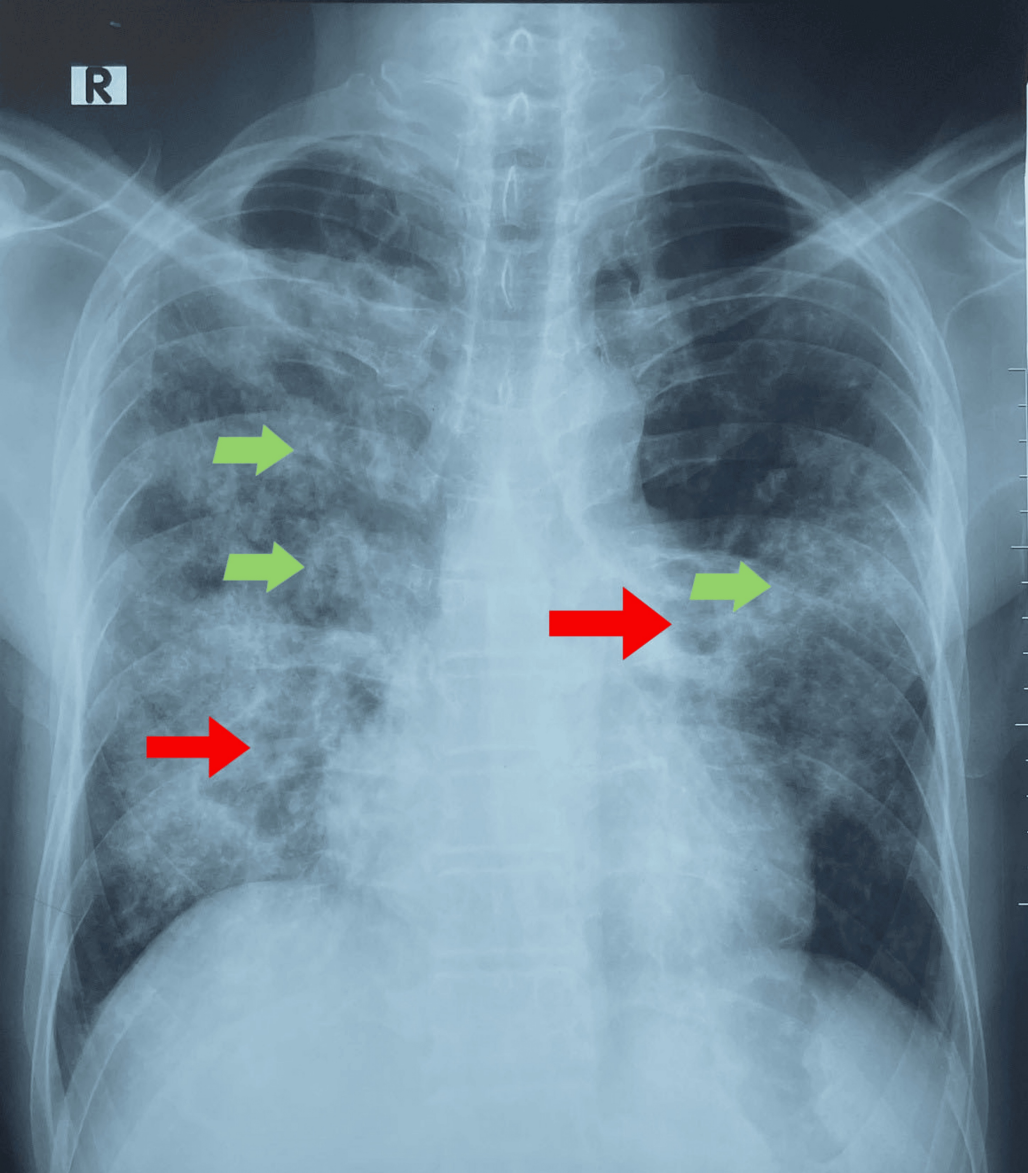
Chest X-ray of the patient X-ray of the chest revealed bilateral (B/L) multiple cysts (red arrows) and nodular lesions (light green arrows).

His Mantoux test was negative. Blood picture showed neutrophil leukocytosis with hypochromic microcytic appearance of RBC. Although the patient complained of a productive cough, only a small amount of sputum was produced. Three samples of sputum were negative for acid-fast bacilli (AFB). His chest X-ray on admission is shown in Figure 1.

Following sputum and blood cultures including TB culture, the patient was started on intravenous (IV) ceftriaxone 1g twice daily for suspected lower respiratory tract infection. Despite treatment, the patient failed to show any significant improvement other than resolution of fever and dropping of CRP to 17 mg/L. His ESR was persistently high at around 60 mm/hr, and he had generalized malaise, loss of appetite, and arthralgia.

**Figure 2:**
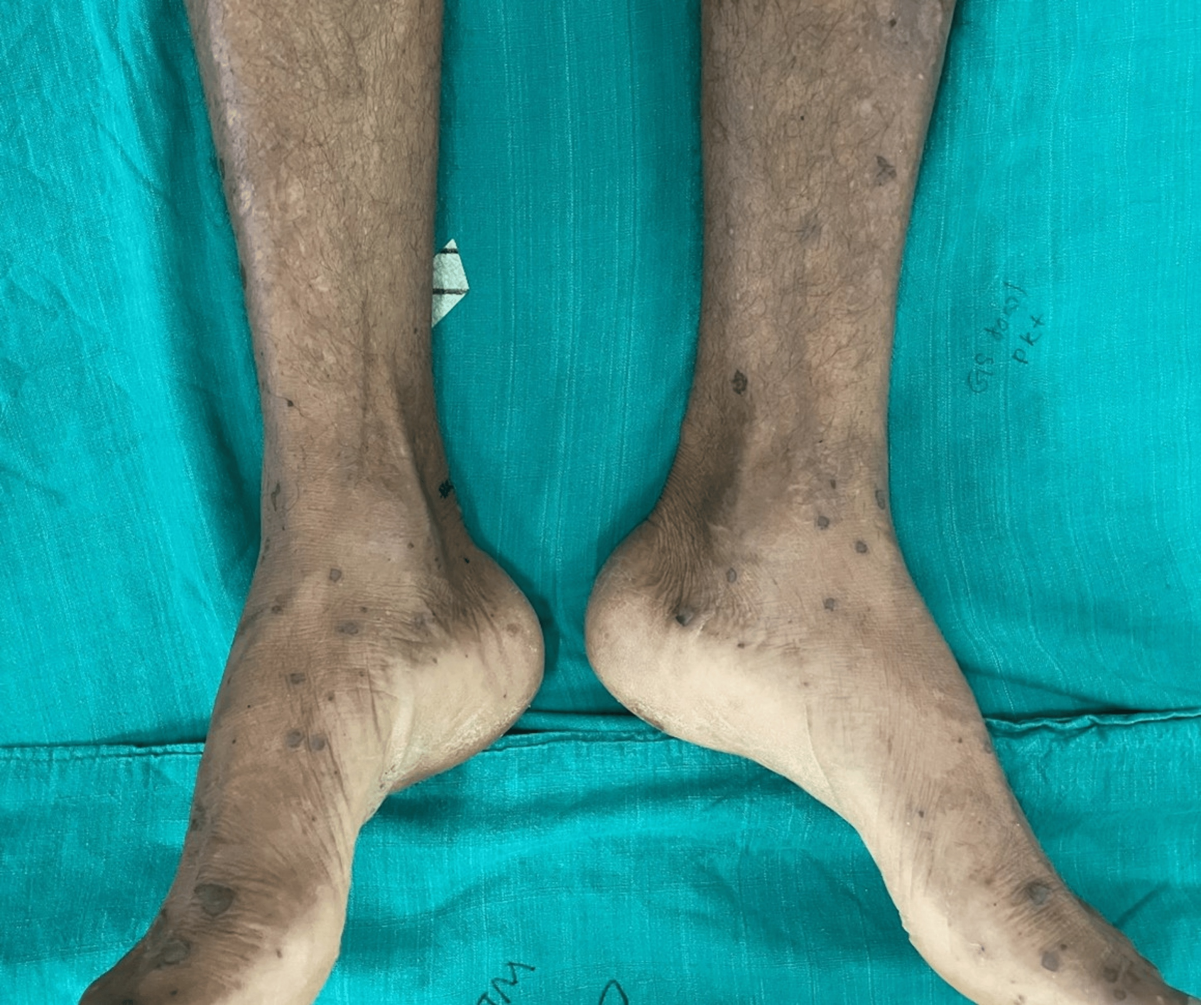
Vasculitic rash

A vasculitic rash appeared on both lower limbs on the fifth day of hospitalization (Figure 2).

Therefore, systemic vasculitis was included in the differential diagnosis, and a vasculitic screening was initiated. Investigation results of his vasculitic screening are presented in Table 2.

**Table 2:** Investigations of vasculitic screening of the patient HPF, high power field

Investigation	Value	Reference range
Urine full report		
Pus cells	1 per HPF	0-5 per HPF
Red cells	4-5 per HPF	Less than 5 per HPF
Serum creatinine	61.8 umol/L	49-115 umol/L
Erythrocyte sedimentation rate	60 mm/hr	Less than 10mm/hr

An examination of the skin biopsy taken at the site of the rash revealed leukocytoclastic vasculitis without granulomas of necrosis, indicating a nonspecific finding.

Due to the diagnostic uncertainty surrounding TB and vasculitis, collaborative discussions were held between the rheumatologist and chest physician. As a result of negative TB studies, persistently elevated ESR, involvement of the respiratory tract with hemoptysis, vasculitic rash, and the presence of red blood cells in urine analysis, GPA was considered to be a high possibility.

A bronchoscopy was performed to obtain samples, which revealed bilaterally inflamed mucosa with healed ulcers, and bronchial washings were collected for AFB, TB GeneXpert, TB culture, and cytology. The cytoplasmic ANCA against proteinase 3 (C-ANCA/PR3) levels were sent for analysis, but perinuclear ANCA (P-ANCA) levels were not measured.

**Figure 3:**
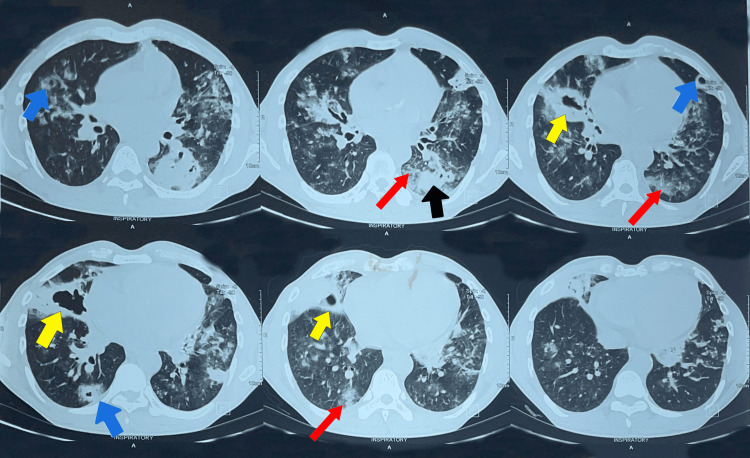
High-resolution CT of the patient There are multiple, irregularly margined lung nodules of variable sizes scattered throughout the lungs (blue and yellow arrows), with some of them demonstrating cavitations (yellow arrows). Multiple patchy areas of consolidations (black arrow) surrounded by ground-glass opacifications (red arrow) are seen scattered in both lungs.

The results of the TB GeneXpert test were negative, effectively ruling out TB. Following the high-resolution computed tomography (HRCT) imaging, features suggestive of GPA were detected, confirming the diagnosis. Figure 3 shows HRCT images demonstrating findings consistent with GPA.

A subsequent C-ANCA/PR3 test revealed a markedly elevated level of more than 200 RU/mL (normal range: less than 20 RU/mL), which further supported the diagnosis of GPA.

According to the latest European League Against Rheumatism/American College of Rheumatology (EULAR/ACR) classification criteria, the patient scored more than five points (endobronchial involvement 2 points, positive C ANCA 5 points, and pulmonary nodules, masses, and cavitation on chest imaging 2 points), confirming the diagnosis of GPA; therefore, it was decided that a renal or lung biopsy was not essential for diagnosis.

Remission was induced with IV methylprednisolone 500 mg daily for three days and cyclophosphamide. For maintenance, the patient was started on azathioprine and oral prednisolone 30 mg once daily. The patient made a remarkable recovery, with resolution of dyspnea, cough, hemoptysis, and healing of the vasculitic rash.

## Discussion

GPA, previously known as Wegener’s granulomatosis, is a rare autoimmune disease primarily affecting small-sized vessels. Dr Friedrich Wegener first described the disease in 1936 as a necrotizing granulomatous inflammation of the upper and lower respiratory tract [1].

Churg and Goodman described the “Wegener's triad” of glomerulonephritis, necrotizing granulomatous inflammation of the respiratory tract, and necrotizing systemic angiitis in 1954 [1]. It is associated with ANCA and can be triggered by a variety of factors, including infection, environmental triggers, drug-induced vasculitis, and genetic predisposition.

The pathogenesis of this disease is thought to be multifactorial, and the exact mechanism is not yet elucidated. It is believed that C-ANCA causes neutrophil-mediated vascular injury by acting against a protein called proteinase 3 (PR3) [5].

GPA typically causes symptoms in the upper and lower respiratory tract, accounting for up to more than 70% of cases [2,6]. The most common symptoms reported are recurrent sinusitis, epistaxis, oral ulcers, otitis media, cough, hemoptysis, and dyspnea. The involvement of the renal system in GPA is also common. Patients with GPA usually present with a prodrome of nonspecific symptoms, including fever, fatigue, weight loss, myalgia, and arthralgia, preceding the appearance of organ-specific symptoms.

TB is a major differential diagnosis for GPA, especially in TB-prevalent areas. The considerable overlap between the features of GPA and TB, such as prodromal symptoms of fever, malaise, loss of appetite, and weight loss, combined with similar respiratory manifestations such as cough and hemoptysis and high ESR, makes it difficult to differentiate between GPA and TB at the onset of illness.

TB can coexist with GPA and can potentially trigger ANCA-associated vasculitis. However, before treating GPA, it is important to accurately exclude TB due to the possibility that corticosteroids, the mainstay of GPA therapy, may exacerbate TB infection. Since our patient's symptoms continued to deteriorate, it was urgent to confirm the diagnosis of GPA to initiate appropriate treatment.

Due to the prolonged period required for TB cultures to yield results, waiting for confirmation posed a significant challenge. Since the patient was not producing adequate sputum despite nebulization therapy, a bronchoscopy was performed to obtain diagnostic samples.

Although both GPA and TB can cause skin involvement, their respective manifestations are often different. GPA is characterized by skin manifestations related to small vessel vasculitis, such as palpable purpura, nodules, ulcers, or livedo reticularis. In contrast, TB may cause erythema nodosum and lupus vulgaris, which are often the result of immune-mediated reactions or direct involvement of the organism. However, TB can also present with palpable purpura, blisters, and ulcers due to secondary small vessel vasculitis [6].

It is important to recognize these differences in skin manifestations to be able to differentiate between GPA and TB, as was the case in our patient.

HRCT findings of Wegener's granulomatosis range from nodules and masses to ground-glass opacity and lung consolidation. The most common manifestation of Wegener's granulomatosis is lung nodules, which affect 40-70% of patients [7,8]. However, TB and GPA can have similar radiological findings, which complicates the diagnosis. Cavitatory and nodular lesions, for instance, can be observed in both conditions, and necrotic lesions in the lungs of patients with GPA have radiological similarities to the caseous necrosis typically observed in TB patients. The presence of alveolar hemorrhages, particularly in GPA, may assist in differentiating between the two, thus facilitating a more accurate diagnosis of GPA over TB, as in our patient [9].

Histologically, GPA is characterized by necrotizing noncaseating granulomatous vasculitis, which helps differentiate it from TB-associated vasculitis. However, this is not a uniform finding. There can be non-specific chronic inflammation in some cases, resulting in lesions that are similar to those seen in TB. This was the case in our patient.

Classical autoantibodies for diagnosing GPA may also be positive in cases of TB [1]. Therefore, caution should be taken when interpreting the results, particularly when there is a possibility of TB. In around 80-90% of patients with active GPA and approximately 50% of those with inactive disease, positive C-ANCA/PR3 results are reported [10]. TB has the capability to induce ANCA, posing a diagnostic dilemma in the context of GPA.

ANCA has been reported in around 44% of patients with TB [10]. In addition, studies have demonstrated increased detection of ANCA following TB treatment compared with pre-treatment, suggesting that TB treatment itself can induce the de novo formation of ANCA [10]. P-ANCA appears to be most commonly associated with TB, while C-ANCA/PR3 is commonly associated with GPA, which can be useful in distinguishing GPA from TB.

TB can result in the production of ANCA that target bactericidal permeability-increasing proteins (BPI). These ANCAs were detected through the other immunological tests that we performed. In such cases, and in P-ANCA or C-ANCA positive cases, in the context of TB, enzyme-linked immunosorbent assay (ELISA) can identify the presence and quantity of ANCA against PR3, which is specific to GPA. A combination of indirect immunofluorescence (IIF) and ELISA results indicates 99% specificity for ANCA-associated vasculitis and 73% sensitivity for GPA [11]. Therefore, combining IIF and antigen-specific ELISA can provide valuable insight into diagnosing GPA and TB.

Biopsy is the gold standard for the diagnosis of GPA. Renal biopsy, the most common approach, usually reveals nonspecific glomerulonephritis. A lung biopsy may demonstrate granulomatous necrotizing vasculitis of small vessels.

In summary, differentiation between GPA and TB should be based on a combination of clinical, immunological, radiological, and histopathological findings.

The EULAR/ACR has released the classification criteria for GPA that integrate clinical, serological, and histopathological findings, as outlined in Table 3.

**Table 3:** EULAR/ACR classification criteria for GPA *Indicates features in our patient C-ANCA, cytoplasmic antineutrophil cytoplasmic antibodies; EULAR/ACR, European League Against Rheumatism/American College of Rheumatology; GPA, granulomatosis with polyangiitis; P-ANCA, perinuclear antineutrophil cytoplasmic antibodies; TB, tuberculosis

Criteria	Score
Clinical criteria	
Nasal involvement: bloody discharge, ulcers, crusting, congestion, blockage, or septal defect/perforation	+3
Cartilaginous involvement: inflammation of ear or nose cartilage, hoarse voice or stridor, endobronchial involvement,* or saddle nose deformity)	+2
Conductive or sensorineural hearing loss	+1
Laboratory, imaging, and biopsy criteria	
Positive test for C-ANCA or anti-PR3 antibodies*	+5
Pulmonary nodules, mass, or cavitation on chest imaging*	+2
Granuloma, extravascular granulomatous inflammation, or giant cells on biopsy	+2
Inflammation, consolidation, effusion of the nasal/paranasal sinuses, or mastoiditis on imaging	+1
Pauci-immune glomerulonephritis on biopsy	+1
Positive test for P-ANCA or anti-myeloperoxidase (MPO) antibodies	-1
Blood eosinophil count more than or equal 1x10^9^/liter	-4

Diagnosis of GPA can be made if the cumulative score is more than 5 points. Our patient had a total of 9 points. A summary of the comparison between TB and GPA is illustrated in Table 4.

**Table 4:** Summary of the comparison between TB and GPA AFB, acid-fast bacilli; C-ANCA, cytoplasmic antineutrophil cytoplasmic antibodies; GPA, granulomatosis with polyangiitis; P-ANCA, perinuclear antineutrophil cytoplasmic antibodies; TB, tuberculosis

	TB	GPA
Cause	Mycobacterium tuberculosis (infectious)	Autoimmune vasculitis (non-infectious)
Fever	Common	Common
Cough	Common (often productive, may have hemoptysis)	Common (often dry, may have hemoptysis)
Hemoptysis	Common in pulmonary TB	Can occur due to pulmonary capillaritis or cavitating nodules
Weight loss	Common	Common
Night sweats	Common	Uncommon
Nasal symptoms	Rare	Common (chronic sinusitis, epistaxis, nasal crusting, saddle nose)
Skin lesions	Rare (erythema nodosum, lupus vulgaris)	Common (palpable purpura, vasculitic rash)
Renal involvement	Rare in primary TB, except in genitourinary TB	Common (rapidly progressive glomerulonephritis)
Chest X-ray findings	Cavitations, infiltrates, lymphadenopathy, miliary pattern	Nodules, cavitary lesions, infiltrates, alveolar hemorrhage
Sputum AFB	Positive in pulmonary TB	Negative
Mantoux test	Usually positive	Negative
ANCA	Usually negative, P-ANCA can be seen after TB treatment	Positive (commonly C-ANCA/PR3)
Biopsy findings	Caseating granulomas	Necrotizing granulomatous inflammation with vasculitis
Response to steroids	Minimal or may worsen disease	Dramatic improvement
Treatment	Anti-TB drugs	Immunosuppressants (steroids, cyclophosphamide, rituximab, azathioprine)

GPA carries a high mortality rate without treatment. Therefore, prompt treatment is essential to improve survival. The aim of treatment is to suppress immune activity. Corticosteroid and steroid-sparing therapy such as cyclophosphamide and rituximab are the mainstay of treatment to induce remission. Maintenance therapy after remission typically lasts 12-24 months or longer and includes azathioprine, methotrexate, and rituximab.

## Conclusions

There are many features of GPA that may overlap with those of TB, creating a diagnostic dilemma. In addition, test results may overlap between the two conditions, complicating the diagnosis. TB should always be considered a possibility in Asian countries due to its high prevalence. Although GPA is relatively uncommon in this region, it should still be considered when there is conclusive evidence against TB, especially since GPA is a treatable condition, and, if left untreated, it carries a high mortality rate. For an accurate diagnosis to be made, a high index of suspicion and comprehensive approach are necessary.
